# Copy Number Variation (CNV): A New Genomic Insight in Horses

**DOI:** 10.3390/ani12111435

**Published:** 2022-06-02

**Authors:** Nora Laseca, Antonio Molina, Mercedes Valera, Alicia Antonini, Sebastián Demyda-Peyrás

**Affiliations:** 1 Departamento of Genética, Universidad de Córdoba, Edificio Gregor Mendel, CN-IV KM396, 14071 Córdoba, Spain; ge2lagan@uco.es (N.L.); ge1moala@uco.es (A.M.); 2 Departamento de Agronomía, ETSIA, Universidad de Sevilla, Ctra Utrera Km 1, 41013 Sevilla, Spain; mvalera@us.es; 3 Departamento de Producción Animal, Facultad de Ciencias Veterinarias, Universidad Nacional de La Plata, La Plata 1900, Argentina; antonini@fcv.unlp.edu.ar; 4 Consejo Nacional de Investigaciones Científicas y Técnicas (CONICET), La Plata 1900, Argentina

**Keywords:** copy number variation regions, functional clustering, SNP genotyping array, horse breed

## Abstract

**Simple Summary:**

This study aimed to contribute to our knowledge of CNVs, a type of genomic marker in equines, by producing, for the first time, a fine-scale characterization of the CNV regions (CNVRs) in the Pura Raza Española horse breed. We found not only the existence of a unique pattern of genomic regions enriched in CNVs in the PRE in comparison with the data available from other breeds but also the incidence of CNVs across the entire genome. Since these regions could affect the structure and dose of the genes involved, we also performed a gene ontology analysis which revealed that most of the genes overlapping in CNVRs were related to the olfactory pathways and immune response.

**Abstract:**

Copy number variations (CNVs) are a new-fangled source of genetic variation that can explain changes in the phenotypes in complex traits and diseases. In recent years, their study has increased in many livestock populations. However, the study and characterization of CNVs in equines is still very limited. Our study aimed to investigate the distribution pattern of CNVs, characterize CNV regions (CNVRs), and identify the biological pathways affected by CNVRs in the Pura Raza Española (PRE) breed. To achieve this, we analyzed high-density SNP genotyping data (670,804 markers) from a large cohort of 654 PRE horses. In total, we identified 19,902 CNV segments and 1007 CNV regions in the whole population. The length of the CNVs ranged from 1.024 kb to 4.55 Mb, while the percentage of the genome covered by CNVs was 4.4%. Interestingly, duplications were more abundant than deletions and mixed CNVRs. In addition, the distribution of CNVs across the chromosomes was not uniform, with ECA12 being the chromosome with the largest percentage of its genome covered (19.2%), while the highest numbers of CNVs were found in ECA20, ECA12, and ECA1. Our results showed that 71.4% of CNVRs contained genes involved in olfactory transduction, olfactory receptor activity, and immune response. Finally, 39.1% of the CNVs detected in our study were unique when compared with CNVRs identified in previous studies. To the best of our knowledge, this is the first attempt to reveal and characterize the CNV landscape in PRE horses, and it contributes to our knowledge of CNVs in equines, thus facilitating the understanding of genetic and phenotypic variations in the species. However, further research is still needed to confirm if the CNVs observed in the PRE are also linked to variations in the specific phenotypical differences in the breed.

## 1. Introduction

Copy number variations (CNVs) are defined as a change in the DNA sequence compared to a reference assembly due to the loss (deletions) or gain (insertions and duplications) of nucleotides bases. CNVs, which usually range from one kilo-base (kb) to several mega-bases (Mb) [1], were associated in livestock animals with changes in the phenotypic expression of simple traits (such as the presence or absence of horns, [2]), and also disease susceptibility and genetic disorders [3]. In addition, recent studies carried out on wildlife and livestock species have pointed to CNVs as a major source of genetic and phenotypic variation among individuals [4,5,6]. For this reason, increasing our knowledge of the existence and function of CNVs in livestock, particularly related to complex traits and environmental adaptability, contributes to a greater genetic improvement of economic and production traits and animal health [7]. Currently, CNVs can be detected using a range of different platforms, including array comparative genome hybridization (aCGH) [8], single nucleotide polymorphism (SNP) arrays [9], and next-generation sequencing (NGS) [10]. Particularly, the availability of SNP array data in large livestock populations genotyped for genomic breeding purposes has led to a considerable improvement in the characterization of the CNV landscape in some livestock species [11,12].

Studies on CNV diversity are relatively novel and scarce in horses. The first report was published by Doan, et al. [13], which suggested that CNVs are common in the horse genome and may modulate the biological processes underlying different traits. Thereafter, several studies have reported the association of CNVs with diseases [9,14,15,16], chromosomal abnormalities [17,18], and phenotypic traits [19,20,21,22], as well as reported CNV regions overlapping with several genes associated with the reproductive system [23] or adaptability to high temperature and humidity [24]. However, the largest study assessing the CNV landscape in several European horse breeds was recently published by Sole, et al. [12] where they identified CNV regions overlapping with QTLs previously associated with changes in fertility, coat color, conformation, and temperament. Although these studies provide a basis to understand the role of CNVs in equine biology, the current information is still insufficient for the efficient discovery of variants affecting the phenotype, and even more, its association with the phenotypes of complex traits.

The Pura Raza Española (PRE) breed, also known as the Andalusian breed, is the most important and widespread horse breed in Spain, with more than 250,000 active individuals. Although 23.3% of its census is distributed over 62 different countries around the world [25], the breeding program is managed worldwide by the Real Asociación Nacional de Criadores de Caballos de Pura Raza Española (ANCCE) from Spain. The PRE horse was recognized as an official breed in the 15th century [26] being considered as a horse of great beauty, with a noble temperament and a great capacity for learning, which explains its success in certain competitions such as dressage, despite being originally selected as a saddle horse. Although a few studies analyzing the genomic landscape of the breed were recently published [17,25,26], to the best of our knowledge, there are no previous studies analyzing CNV variability in this breed.

The aim of this study was therefore to investigate for the first time the distribution pattern of CNVs, characterize CNV regions, and identify biological pathways affected by CNVRs in the PRE horse breed. To achieve this, we analyzed a large cohort of animals genotyped using high-density SNP genotyping technologies. In addition, we attempted to compare the CNV structure of this population with the genome-wide CNVRs identified in other horse breeds, and finally, compare the CNVRs found in different horse breeds.

## 2. Materials and Methods

### 2.1. Sampling, Genomic DNA Isolation, Genotyping, and Quality Control

We selected 805 living individuals from 373 PRE herds showing the present diversity of the population for single nucleotide polymorphism (SNP) genotyping using a range of criteria, including sample availability and low average relatedness among individuals.

Genomic DNA was isolated from blood or hair samples using DNeasy Blood & Tissue Kit (Qiagen, Hilden, Germany), following the manufacturer’s protocol. The DNA was checked for quality and quantity by Nanodrop™ spectrophotometry (ThermoFisher scientific, Madrid, Spain) and gel electrophoresis. All the individuals were genotyped using the 670 K Affymetrix Axiom™ Equine Genotyping Array (ThermoFisher), including uniformly distributed 670,804 markers [27]. Firstly, Axiom Analysis Suite 5.0 software was used to process and filter genotypes based on DishQC (DQC) and call rate (CR) parameters (DQC ≥ 0.82, sample CR ≥ 0.95, and SNP CR ≥ 97), following the *Best Genotyping Practices Workflow* procedure. Only the samples and SNPs which passed the quality control were kept for the following analyses. The final dataset included 552,965 SNPs located on autosomal chromosomes.

### 2.2. CNV Data Analysis

CNV calling was performed using PennCNV v.1.0.5 software [28], based on an integrated hidden Markov model which incorporates multiple sources of information, including relative signal intensities (log R ratio, LRR) and minor allele frequencies (B allele frequency, BAF) per SNP, the distance between adjacent SNPs, the populational frequency of the B allele (PFB), and the GC content of the genomic regions in which each marker is located.

For this, we first extracted the LRR and BAF values per individual and marker from the raw genotyping files (CEL) using the Axiom™ CNV summary tool [29]. Thereafter, we compilated a PFB file by averaging the BAF values of each marker in the whole population, using the compile pfb script included in PennCNV. In addition, we estimated the percentage of GC content in the genomic region surrounding each marker position (±500 kb) using a self-made R script and FASTA information of the EquCab3 horse genome assembly [30] which is employed by the PennCNV algorithm to limit the effect of genomic waves produced by high GC content (according to Diskin, et al. [31]). Finally, we performed individual-based CNV calling using the *-test* option of PennCNV, with the -*gcmodel* and *-pfb* corrections.

CNV filtering and QC analysis were performed using the default PennCNV parameters (standard deviation for LRR  ≤  0.35, BAF drift < 0.01, and waviness factor  ≤ 0.05). Only CNVs larger than 1 kb including at least five consecutive SNPs located on autosomal chromosomes were retained for further analysis since PennCNV calls for the sex chromosomes are unreliable and difficult to interpret (according to the software developer [28]). Finally, to determine the maximum number of CNVs that can be present in an animal for the analysis to be reliable (as proposed by Drobik-Czwarno, et al. [32]), we performed an outlier detection procedure assuming a two-tailed distribution, which determined the exclusion of all the individuals with more than 56 CNV calls (*n* = 151). This procedure was performed since PennCNV software tends to overestimate CNV fragments in individuals in which genotyping quality is not optimal. The final dataset included 654 horses.

### 2.3. Determination of CNV Regions and Gene Annotation

Individual CNV calls overlapping in at least one base pair in at least two animals were concatenated into CNV regions (CNVRs) using HandyCNV software [33]. The CNVRs were classified as gains (duplications), losses (deletions), or mixed CNVRs, in which both deletions and duplications were observed. In addition, we estimated the genomic percentage covered by CNVRs at a chromosome level as the sum of all the CNVRs in a given chromosome in relation to its total length.

Finally, the gene content of the CNVRs was assessed based on EquCab 3.0 as the reference genome using Ensembl Biomart [34]. Functional analysis, including Gene Ontology (GO) and Kyoto Encyclopedia of Genes and Genomes (KEGG) pathways, was established by using DAVID Bioinformatics v6.8 [35] and Uniprot online resources [36]. Finally, all the preliminary findings were confirmed by performing an extensive review of the available literature in public databases.

### 2.4. Comparison of CNVRs with Previous Studies

To determine the existence of differences in the CNV landscape among breeds and populations, we compared our results with eleven previous CNVR reports focused on CNVR characterization in horses. For this, we combined all the CNVRs reported in those studies to generate a large consensus CNVR list which was compared with our findings using HandyCNV software.

## 3. Results and Discussion

### 3.1. Detection of Genome-Wide CNVs

**Figure 1:**
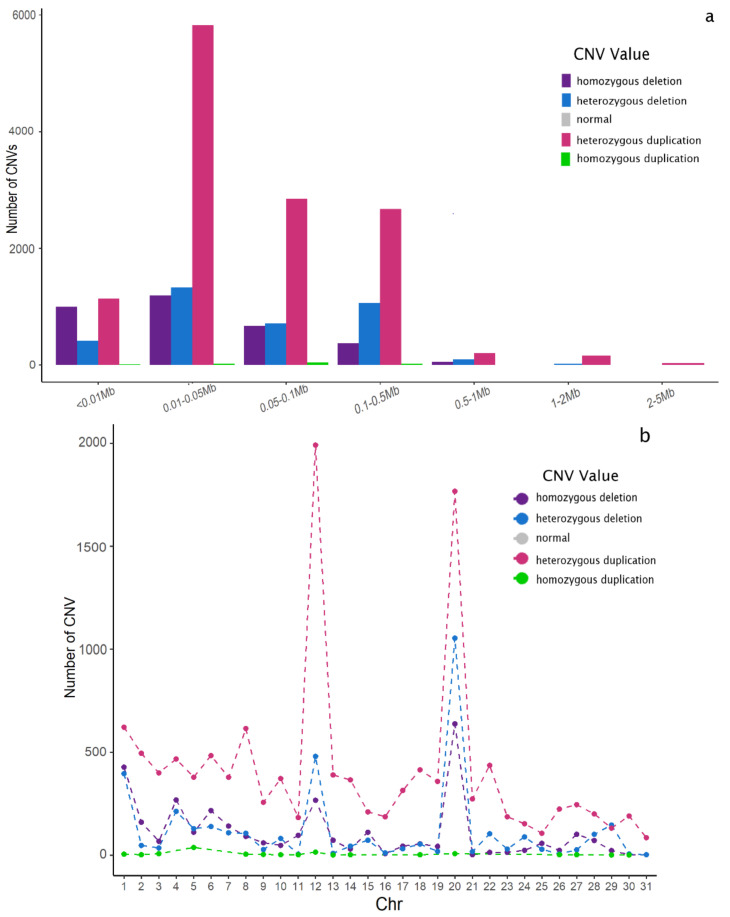
(**a**) Length distribution of CNVs identified; (**b**) chromosomal distribution of CNVs. Number of CNVs present on each chromosome. Purple (homozygous deletion), blue (heterozygous deletion), grey (normal), pink (heterozygous duplication), and green (homozygous duplication).

Advances in the identification of CNVs with a biological function are increasing, since they affect genomic sequences which have been associated with a vital role in the regulation of gene functions by altering gene structure, dosage, and expression (causative variants), thus explaining a large part of the phenotypic variation in several traits and species [1], including the horse [37,38]. However, our knowledge of CNVs that contribute to complex traits and diseases in horses is still limited. In this study, we performed a populational analysis description of the CNV landscape by using high-density genotypes in a large cohort of 654 Pura Raza Español horses, identifying 19,902 segments located across the 31 autosomes. The average number of CNVs per individual was 30.43, ranging from 1.024 kb to 4.55 Mb, with an average of 96.07 kb (Table 1 and Figure 1a). The distribution of the CNVs of each chromosome is shown in Figure 1b; ECA12 and ECA20 being the chromosomes with the highest number of CNVs, with nearly ten times more CNVs than the average values. ECA12 is one of the smallest chromosomes in the horse, and therefore, the increased CNV density detected may suggest that small chromosomes tend to retain a higher number of CNVs. However, ECA29, ECA30, and ECA31 are even smaller than ECA 12, without showing any sign of such an increase in CNV density (in fact, ECA31 was the chromosome showing the lower CNV density). Similarly, ECA20 was the densest chromosome in terms of SNPs analyzed with almost twice the SNPs per Mb than the rest of the genome (≈507 vs. ≈235, respectively), in agreement with the recent findings reported by Rafter, et al. [39] which demonstrated that more CNVs could be detected using high-density than medium-density arrays in cattle. However, a similar SNP density was observed in BTA6 (≈472, Table S1), in which the number of CNVs detected was similar, or even lower, than that observed in the rest of the genome. However, results of BTA12 and BTA20 are consistent with the previous findings reported by several authors in different breeds, in which the prevalence of CNVs in those chromosomes was also high [9,12,14,18,22,40]. Although we did not find a conclusive cause that may explain this peculiarity, it is logical to assume that these regions may carry genes involved in pathways in which a mutation or a change in the genome can provide an evolutive advantage in the species, or at least that they do not have a CNV, in which a duplication or deletion is incompatible with life.

Although the number of deletions and duplications detected differed among chromosomes (Table S1), an interesting finding was the fact that the number of duplications (12,987) exceeded the number of deletions (6915) in most of them, with the sole exception of ECA1, ECA4, and ECA29, in which the opposite pattern was observed. These results are similar to other studies [9,12,13,14,16,24], which reported more gains than losses in several horse populations. Although CNVs can be a source of wide variability at the same locus, duplications are more likely to occur in large CNVs than deletions, since they are more tolerated by the genome since no loss of genetic material occurs [40,41]. This might be since duplications are kept for a long time since the deleted regions involving codifying or regulatory regions tend to be purged across the generations due to the existence of “purifying” selection [42]. For the same reason, it was proposed that duplications located in coding or enhancing sequences could increase the genetic diversity in the organisms, thus contributing to phenotypic variation and the putative ability to thrive in adverse environments [43,44]. However, the loss of genetic material due to deletions might play a significant role in the genetics of complex traits, even though this has not been directly observed in several gene mapping studies [42]. Still, it is worth mentioning that opposite results have also been reported in horses [19,22,40], although in those studies, results may be explained by the use of different sequencing and genotyping platforms (medium-density arrays) during CNV detection, as well as by the scarce number of individuals analyzed, as demonstrated by Pawlina-Tyszko, et al. [16], Metzger, et al. [19], and Kader, et al. [20]. In this context, Di Gerlando, et al. [5] and Rafter, Gormley, Parnell, Kearney and Berry [39] demonstrated the importance of array density as a factor affecting the discovery of CNVs in sheep and cattle, high-density SNP arrays being associated with better resolution and sensitivity in the CNV detection. On the contrary, Sole, et al. [12] obtained similar results than us in terms of duplication/deletion ratios by analyzing nearly 1800 horses using the same HD SNP array employed in the present study. It is therefore important to mention that comparisons between studies involving CNV detection using different methodologies and algorithms should be made with caution.

### 3.2. Determination of CNV Regions

**Figure 2:**
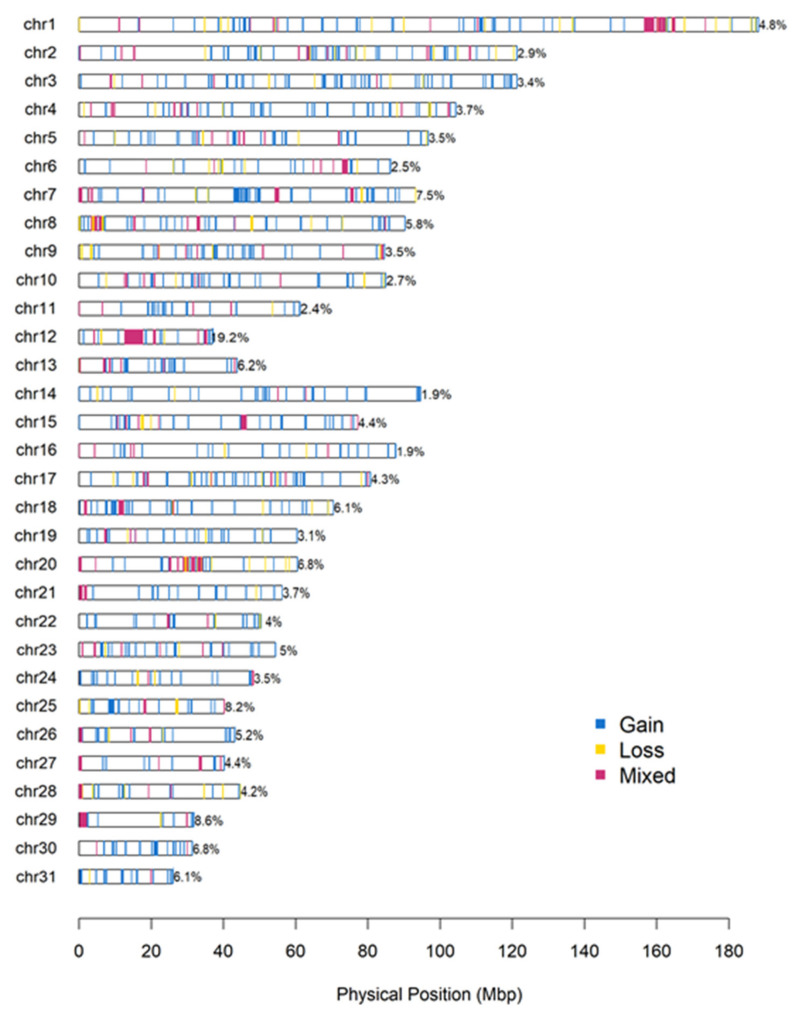
Map of CNVRs in the 31 equine autosome chromosomes. Blue, yellow, and red represent gain, loss, and mixed, respectively.

The overlapping CNVs in at least one base pair in at least two samples allowed us to detect 1007 CNVRs, including 694 gains, 139 losses, and 174 mixed regions (Table 2 and Table S2), covering 99.79 Mb (representing 4.4% of the genome). Among these, 109 (31 duplications, 19 deletions, and 59 mixed) were present in at least 5% of the PRE population analyzed. These results are higher than those obtained in several studies reported to date in other equine breeds, which range from 0.6 to 3.7% [13,14,20,21,22,23,24], but lower than those obtained by Schurink, et al. [9] and Metzger, et al. [19] using PennCNV software. In addition, although several CNVRs were observed in all the chromosomes (Figure 2), the coverage of CNVRs in each chromosome varied from 1.9% in ECA14 and ECA16 to 19.2% in ECA12. These results in terms of variability agree with most of the previous studies carried out on horses [9,12,13,14,16,19,20,21,22,23,24]. However, several of them reported that ECA12 is particularly enriched in CNVRs, including a cluster of genes associated with the development of olfactory receptors (ORs) [13,18,19]. In all of them, it has been suggested that these genes have undergone selection, or have even increased the number of copies, through CNV gain and loss processes during the domestication of the horse. However, it is worth mentioning that the overrepresentation of these OR genes in CNV regions is not only present in horses but also humans [45], cattle [46,47], pigs [48], and sheep [49].

### 3.3. Comparative Analysis with Previously Known CNVRs

Differences in CNVRs have been successfully employed as a method of differentiation of horse breeds [12], since they allow us to find variants and regions which may be associated with its historical evolution, adaptability to a specific environment, or an improvement in the phenotype in traits of interest, such as the grey coat [50]. For this reason, we compared CNVRs identified in this study with those previously reported in eleven different reports [9,12,13,14,16,19,20,21,23,24] by generating a consensus list including all the different CNVRs reported previously (*n* = 8292; Table S3). Our analysis showed that 61% (614) of the CNVRs which we detected in PRE overlapped with some of these, whereas near 40% were described for the first time in this study. In terms of variability, this large difference detected among breeds supports the hypothesis that CNVRs can be used as a reliable genetic footprint to discriminate among breeds, as proposed by Sole, Ablondi, Binzer-Panchal, Velie, Hollfelder, Buys, Ducro, Francois, Janssens, Schurink, Viklund, Eriksson, Isaksson, Kultima, Mikko and Lindgren [12]. However, it must be taken into account that the detection methodology, the number of samples analyzed, the genotyping platform, and the criteria for searching for CNVRs employed in each study were different, and therefore, further research is still in need for a proper validation in the species. However, it was a highly interesting finding that most of the CNVR losses (95.7%), in contrast to only 63.6% of the CNVR gains, overlapped with previous CNVR reports, although it also may be explained by the fact that in most of the studies, the number of losses was higher than the number of gains, conversely to our findings. On the other hand, as we mentioned before, our analysis included 393 newly described CNVRs. This percentage agrees with Sole, et al. [12], who reported a proportion of unique CNVs ranging from 30–50% by comparing Draught and Warmblood horses, which was lower than that reported by Schurink, et al. [9] in Friesian horses (58%). Interestingly, 22 of the unique regions detected in this study were quite common within the PRE population, being present in at least 5% of the horses analyzed. These findings also agree with Sole, et al. [12], who demonstrated the existing differences among the frequencies of common CNVRs between breeds.

Finally, we reviewed the CNVRs found in a total of 38 different breeds of horses in previous studies (Table S4). As we expected, the number of individuals analyzed was positively correlated with the number of CNVRs found (r^2^ = 0.45). However, this value suggests the existence of a racial component since the correlation was moderate. For example, previous CNVR reports ranged from 1 in Brandenburger (2 individuals analyzed) to 5350 in Friesian (222 individuals analyzed), the breeds with more CNVR per individual being the Friesian, Vlaams paard, German draft, and Ardenner horses. In contrast, Warmbloods and Swedish warmblood were the breeds with the fewest CNVRs per individual, without taking into account the breeds in which the CNVRs were analyzed by consensus between methodologies [19]. Paradoxically, the Friesian breed was analyzed in two studies [9,12] and the number of CNVRs found was very different, probably as a result of the different methodologies used. Although most of the CNVs studies performed in the different breeds were performed using PennCNV, several different approaches and software (such as CNVRuler [51] or BEDTools [52]) were used to overlap the CNVs into CNV regions. Drawing conclusions from this comparison is extremely complex because the differences observed could be attributed to all the factors affecting the CNV call, but also in this case, to the use of different methodologies applied for CNVR discovery.

### 3.4. Functional Annotations of CNV Regions

CNVRs are involved in modulating the gene function in multiple ways, including changing gene structure, altering gene regulation, and exposing recessive alleles. For this reason, we performed functional analysis on the genes affected by a CNVR to understand its potential effects on biological processes in horses. Interestingly, 71.4% of the CNVRs contained genes (2105) (Table S5), among which 77.86% were protein-coding genes, 15.53% were long noncoding RNAs (lncRNAs), 2.94% were pseudogenes, 1.66% were microRNAs, 1.05% were small nuclear RNAs (snRNAs), and 0.76% were small nucleolar RNAs (snoRNAs). These results are similar to other studies in horses, indicating that a high percentage of CNVRs cover genomic regions involved in genes. For instance, Ghosh, et al. [23] found that 82% of the CNVRs identified concatenated with one or more genes. Similarly, other studies have reported that 80% [15], 79.3% [12], 69.7% [20], and 49.2% [9] of the CNVRs found involved genes.

Functional annotation analysis revealed that the most significantly enriched biological processes were included in three main categories: olfactory receptor activity (*p* Benjamini = 5.3 × 10^−135^), G-protein coupled receptor activity (*p* Benjamini = 3.8 × 10^−107^), and immune response (*p* Benjamini = 9.9 × 10^−20^) (Table S6). As expected, the KEGG pathway analyses indicated that olfactory transduction was the most affected pathway (*p* Benjamini = 2.7 × 10^−80^), with 311 genes involved. These olfactory system genes are essential for detecting, encoding, and processing chemo-stimuli that can carry information that is important for survival, social interactions, reproduction, and adaptation to the animal environment [53]. In this context, Palouzier-Paulignan, et al. [54] suggested that olfactory receptors are also involved in mammalian appetite regulation and feeding efficiency, and therefore, may be related to food intake. Here, our results agree with Hughes, et al. [55], who proposed that this large family of genes has undergone extensive expansion and contraction through duplication and pseudogenization, giving rise to new functionalities. In addition, several genes related to G-protein coupled receptor activity (358), immune response (66), steroid hormone biosynthesis (14), secondary metabolite biosynthesis, transport, and catabolism (10), and ovum development (5) were located within or nearby CNVRs, which revealed six different functional-term clusters that were significantly enriched (enrichment score higher than 4.76; Table 3). The ontology analysis that we performed agree with previous CNV studies in horses [9,14,19,22] that have identified the olfactory receptors and immunity-related genes as CNV hotspots. Furthermore, Young, et al. [45] provided evidence that OR enrichment in CNVs is not due to positive selection but to the frequent appearance of these genes in segmentally duplicated regions, and that the purifying selection against CNVs is lower in OR-containing regions than in regions containing essential genes [41].

## 4. Conclusions

This study investigated for the first time the distribution pattern of CNVs and CNV regions in the Pura Raza Española horse breed. Our results revealed that a considerably large proportion of the genome (4.4%) was affected by CNVRs, although its distribution among the chromosomes was not uniform. Moreover, we found 394 CNVRs that had not yet been identified in different horse breeds, which may have contributed to the establishment of the PRE phenotype. Finally, functional annotation analysis of CNVRs revealed significant enrichment in genes related to olfactory transduction, olfactory receptor activity, and immune response, pointing to CNVs as hotspots for these genes. This study contributes to our knowledge of CNVs in the equine species and our understanding of genetic and phenotypic variations in the equine genome, but future research is needed to confirm if the observed CNVRs are also linked to phenotypical differences in complex traits.

## Figures and Tables

**Table 1:** Summary of CNVs identified in Pura Raza Española breed.

CNV Type	CNVs *n*	Average Length (bp)	Min Length (bp)	Max Length (bp)
Homozygous deletion	3291	55,077	1150	1,098,544
Heterozygous deletion	3624	107,377	1063	3,209,464
Heterozygous duplication	12,886	103,367	1024	4,552,372
Homozygous duplication	101	95,655	2700	897,981

**Table 2:** Summary of regions CNVs in Pura Raza Española breed.

CNVR Type	CNVRs *n*	Average Length (bp)	Min Length (bp)	Max Length (bp)	Total Length (bp)
Gains	694	78,815	1458	1,169,661	54,697,934
Losses	139	44,882	1063	635,203	6,238,703
Mixed	174	223,309	5458	4,921,979	38,855,746

**Table 3:** Significantly enriched annotation clusters and functional terms.

Functional Cluster (Enrichment Score)	Category	Term	Genes	*p* Value	P Benjamini
Cluster 1 (94.64)	GOTERM_MF_DIRECT	Olfactory receptor activity	355	6.9 × 10^−138^	5.3 × 10^−135^
	INTERPRO	Olfactory receptor	355	2.1 × 10^−131^	3.1 × 10^−128^
	GOTERM_MF_DIRECT	G-protein coupled receptor activity	358	9.8 × 10^−110^	3.8 × 10^−107^
	UP_SEQ_FEATURE	DOMAIN:G_PROTEIN_RECEP_F1_2	362	5.6 × 10^−99^	3.7 × 10^−96^
	UP_KW_BIOLOGICAL_PROCESS	Olfaction	282	5.0 × 10^−97^	4.2 × 10^−95^
	INTERPRO	GPCR, rhodopsin-like, 7TM	362	9.0 × 10^−97^	6.8 × 10^−94^
	INTERPRO	G protein-coupled receptor, rhodopsin-like	348	4.5 × 10^−93^	2.3 × 10^−90^
	UP_KW_BIOLOGICAL_PROCESS	Sensory transduction	284	5.6 × 10^−93^	2.4 × 10^−91^
	UP_KW_MOLECULAR_FUNCTION	Transducer	368	4.4 × 10^−84^	3.0 × 10^−82^
	KEGG_PATHWAY	Olfactory transduction	311	8.6 × 10^−83^	2.7 × 10^−80^
	UP_KW_MOLECULAR_FUNCTION	Receptor	372	2.2 × 10^−65^	7.4 × 10^−64^
	UP_KW_CELLULAR_COMPONENT	Cell membrane	311	2.8 × 10^−54^	1.2 × 10^−52^
Cluster 2 (40.74)	UP_SEQ_FEATURE	DOMAIN:Ig-like	192	8.8 × 10^−44^	2.9 × 10^−41^
	INTERPRO	Immunoglobulin-like domain	192	3.1 × 10^−42^	1.2 × 10^−39^
	INTERPRO	Immunoglobulin-like fold	233	2.2 × 10^−38^	5.6 × 10^−36^
Cluster 3 (30.68)	GOTERM_CC_DIRECT	Integral component of membrane	662	3.7 × 10^−38^	1.8 × 10^−35^
	UP_SEQ_FEATURE	TRANSMEM:Helical	697	2.9 × 10^−31^	6.3 × 10^−29^
	UP_KW_CELLULAR_COMPONENT	Membrane	730	8.5 × 10^−25^	1.8 × 10^−23^
Cluster 4 (8.14)	GOTERM_BP_DIRECT	Phagocytosis, recognition	21	4.5 × 10^−11^	2.6 × 10^−8^
	GOTERM_BP_DIRECT	Phagocytosis, engulfment	21	2.2 × 10^−10^	9.9 × 10^−8^
	GOTERM_BP_DIRECT	Positive regulation of B cell activation	19	6.8 × 10^−10^	2.2 × 10^−7^
	GOTERM_CC_DIRECT	Immunoglobulin complex, circulating	19	2.9 × 10^−9^	4.6 × 10^−7^
	GOTERM_BP_DIRECT	B cell receptor signaling pathway	22	1.2 × 10^−8^	3.4 × 10^−6^
	GOTERM_MF_DIRECT	Immunoglobulin receptor binding	19	1.4 × 10^−8^	2.6 × 10^−6^
	GOTERM_MF_DIRECT	Antigen binding	20	1.9 × 10^−8^	2.9 × 10^−6^
	GOTERM_BP_DIRECT	Complement activation, classical pathway	20	2.0 × 10^−8^	5.2 × 10^−6^
	GOTERM_BP_DIRECT	Defense response to bacterium	24	4.3 × 10^−5^	4.5 × 10^−3^
Cluster 5 (6.54)	UP_SEQ_FEATURE	DOMAIN:BPI1	12	1.1 × 10^−7^	1.5 × 10^−5^
	INTERPRO	Lipid-binding serum glycoprotein, N-terminal	12	4.6 × 10^−7^	5.3 × 10^−5^
	INTERPRO	Bactericidal permeability-increasing protein, alpha/beta domain	12	4.6 × 10^−7^	5.3 × 10^−5^
Cluster 6 (4.76)	KEGG_PATHWAY	Graft-versus-host disease	22	9.1 × 10^−7^	9.4 × 10^−5^
	KEGG_PATHWAY	Type I diabetes mellitus	20	2.6 × 10^−5^	1.3 × 10^−3^
	KEGG_PATHWAY	Allograft rejection	17	2.2 × 10^−4^	8.6 × 10^−3−^

## Data Availability

The data that support the findings of this study are available from the Real Asociación Nacional de Criadores de Caballos de Pura Raza Española (ANCCE). Restrictions apply to the availability of these data, which were used under license for this study. The data are available from the authors with the permission of ANCCE. The data reviewed in this study were obtained from public databases.

## Supplementary Materials

The following supporting information can be downloaded at: https://www.mdpi.com/article/10.3390/ani12111435/s1, Table S1: Number of CNVs (deletions and duplications) identified on the different chromosomes. Table S2: List of CNVRs found in the analyzed PRE horses. Table S3: Combination of all CNVRs found in previously published studies of horses. Table S4: Summary of the number of CNVRs identified in each breed of horse, previously published. Table S5: List of genes found and/or overlapping with CNV regions in PRE horse breed. Table S6: Functional annotation analysis performed using DAVID Database.
